# Measurement and structural invariance of the social determinants of health questionnaire (SDH-Q) across countries

**DOI:** 10.1371/journal.pone.0356884

**Published:** 2026-08-27

**Authors:** Abdulwali Sabo, Garry Kuan, Sarimah Abdullah, Hue San Kuay, Mohammed Dauda Goni, Yee Cheng Kueh

**Affiliations:** 1 Biostatistics and Research Methodology Unit, School of Medical Sciences, Universiti Sains Malaysia, Kubang Kerian, Kelantan, Malaysia; 2 Department of Public and Environmental Health, Faculty of Basic Medical Sciences, Federal University Dutse, Dutse, Jigawa State, Nigeria; 3 Exercise and Sports Science Programme, School of Health Sciences, Universiti Sains Malaysia, Kubang Kerian, Kelantan, Malaysia; 4 Department of Psychiatry, School of Medical Sciences, Universiti Sains Malaysia, Kubang Kerian, Kelantan, Malaysia; 5 Faculty of Veterinary Medicine, Universiti Malaysia Kelantan, Pengkalan Chepa, Kelantan, Malaysia; 6 Institute for Artificial Intelligence and Big Data, Universiti Malaysia Kelantan, Kota Bharu, Kelantan, Malaysia; Bangor University, UNITED KINGDOM OF GREAT BRITAIN AND NORTHERN IRELAND

## Abstract

**Background:**

Social determinants of health (SDH) refer to the social factors and processes that influence health outcomes and contribute to inequalities among different populations. With growing interest in assessing how people perceive these determinants within their communities, this study aims to examine the measurement and structural invariance of the Social Determinants of Health Questionnaire (SDH-Q) across countries.

**Method:**

The study was a cross-sectional survey among 860 undergraduate students from Malaysia and Nigeria (430 each). The convenience sampling method was used in selecting the study participants. Confirmatory factor analysis (CFA) and invariance tests were performed on the SDH-Q model across the countries. Other measures computed were composite reliability (CR), internal consistency based on Cronbach’s alpha, average variance extracted (AVE), and test-retest reliabilities using intraclass correlation coefficient (ICC).

**Result:**

The overall CFA results of the final model with 2 factors and 20 items were satisfactory (CFI = 0.936, TLI = 0.918, SRMR = 0.044, RMSEA = 0.053). The CR of the final SDH-Q model (Model 2) were 0.839 and 0.842 for structural determinants and intermediary determinants, respectively. The overall Cronbach’s alpha value was 0.951 and 0.902 for the Malaysian and Nigerian samples, respectively. Further, the findings demonstrated acceptable measurement and structural invariance across the countries for the Malaysian model (CFI = 0.928, TLI = 0.910, SRMR = 0.051, RMSEA = 0.055) and the Nigerian model (CFI = 0.935, TLI = 0.921, SRMR = 0.048, RMSEA = 0.052).

**Conclusion:**

The SDH-Q with two factors and 20 items is shown to possess acceptable psychometric properties among university students in Malaysia and Nigeria. It can be employed to make valid comparisons across the countries.

## Introduction

The World Health Organization defines the social determinants of health (SDH) as the conditions under which individuals are born, grow, live, work, and age [1]. These conditions are shaped by the political, social, and economic factors [1]. The quality, availability, and equitable distribution of these resources play a major role in shaping a population’s health and well-being. As such, these resources include educational opportunities, safe housing, nutritious food, employment, and access to healthcare [1–4]. The circumstances that are unfavourable to humans often arise from a combination of inadequate policies and programs, inequitable economic systems, and weak governance [2,3]. The socio-political and economic systems of any society should make sure that everyone has fair access to various social resources [2,3].

The term “SDH” reflects a dual meaning. It refers to the social conditions that shape the health of individuals and populations as well as the social processes that lead to the unequal distribution of those conditions among groups with different social class positions [5,6]. Consequently, SDH comprises both the determinants that influence health outcomes and the determinants that cause health inequities [5,6]. The concept of adequate SDH refers to improving the social conditions that affect health and ensuring they are distributed equitably within every society. For this reason, several researchers have proposed using the phrase “social determinants of health and related inequalities” to more accurately capture both aspects, which covers the factors that impact health and the factors that promote health disparities [7–10].

There is an increasing demand for valid and reliable tools that can evaluate individuals’ perceptions towards available SDH at the societal and community level by researchers and healthcare providers [11,12]. Most of the available SDH questionnaires focus on only one dimension of SDH, such as food insecurity and housing [4,13], health literacy [14], social support [15], sexual abuse [16], and social support for physical activity [17]. The systematic review by O’Brien [11] reported that only four existing questionnaires assess multiple SDH domains, including the Income, Housing, Education, Legal Status, Literacy, and Personal Safety (IHELLP) Questionnaire; the Questionnaire Literacy Screen; the We Care tool; and the Child Poverty Tool and Resource Guide. However, these questionnaires fail to account for some crucial aspects of SDH, such as social support, childhood circumstances, access to healthcare, and other essential resources. Moreover, most of these tools are long-form and require a lot of time to complete, making their usage limited in many healthcare environments where brief and valid screening tools are needed [18–20].

A brief self-report tool for assessing SDH, namely, the Steps to Better Health Questionnaire (STBH-Q), was previously developed and validated among the Australian adult population [18]. The STBH-Q consists of 16 items under five domains, designed to evaluate multiple dimensions of SDH. However, the study had reported some limitations, including several item cross-loadings during the exploratory factor analysis (EFA) and some factors that had only two items [18]. To address these concerns, Sabo et al., [21] developed a new, concise SDH measure (SDH-Q) grounded in the CSDH framework [1]. The SDH-Q includes two dimensions, namely the structural determinants (10 items) and intermediary determinants (10 items), and demonstrated adequate psychometric properties while overcoming the limitations of earlier instruments [21]. Nonetheless, the study was conducted solely among Nigerian undergraduate students. Therefore, the present study aims to evaluate the measurement and structural invariance of the SDH-Q among university students in Malaysia and Nigeria.

Testing the validity of the SDH questionnaire across Nigeria and Malaysia is grounded in the cross-cultural holistic health approach, which emphasizes the need to understand health within diverse social, cultural, and contextual settings. Furthermore, the United Nations emphasizes the need to understand health disparities among diverse population groups, improve health outcomes, and promote community engagement through context-specific and culturally appropriate health approaches [22]. This aligns with the objectives of Sustainable Development Goal (SDG) 3, which focuses on ensuring healthy lives and promoting well-being for all, as well as SDG 10, which aims to address disparities within and among populations [22]. Therefore, cross-cultural validation of the SDH questionnaire will provide a valuable framework for accurately assessing health inequalities and informing the development of evidence-based interventions that are sensitive to the unique social and cultural realities of Nigeria and Malaysia.

Although Malaysia and Nigeria are geographically distant, they share several socioeconomic and developmental similarities. Malaysia is recognized as one of Asia’s leading economies, while Nigeria is the largest economy in Africa. These similarities, alongside their distinct regional contexts, make comparative research between the two countries relevant for gaining deeper insights into the SDH across diverse populations. Previous research by Abbas and Karage [23] demonstrated that healthcare and safety reforms have contributed to reducing disparities between advantaged and disadvantaged groups in access to affordable healthcare in both countries; however, the impact of these reforms was reported to be higher in Malaysia than in Nigeria. Against this background, the present study aims to assess the measurement and structural invariance of the SDH-Q among university students in Malaysia and Nigeria, thereby determining its applicability across different cultural and national settings.

## Materials and methods

### Study design

In the present study we employed a cross-sectional study design to recruit a total of 860 undergraduate students, 430 from Universiti Sains Malaysia (USM), Health Campus, and 430 from the College of Medicine and Allied Medical Sciences at Federal University Dutse (FUD), Nigeria. Participants were selected using the convenience sampling approach between December 3 and April 1, 2025. The data collection was done via an online Google Forms survey, which was shared with students through their email, WhatsApp groups, and Telegram. The survey link provided a brief introduction on the study aims and inclusion criteria. Students who consented and met the criteria accessed the link and submitted their responses. This online survey allowed for efficient data collection and supports future research efforts [24]. In addition, we applied convenience sampling because of its practicality, ease of implementation, and usefulness in generating data that can reflect the target population when random sampling is not feasible [25].

### Measures

The measurement scale included two sections: the participants’ general characteristics (age, gender, year of study, field of study, and ethnicity) and the Social Determinants of Health Questionnaire (SDH-Q). The SDH-Q consists of 20 items under two domains: structural determinants of SDH (10 items) and intermediary determinants of SDH (10 items).

The items under the structural determinants domain reflect the factors that shape or reinforce social stratification and define socioeconomic position, using a Likert rating scale from 1 (totally unsatisfied) to 5 (totally satisfied). Items under the intermediary determinants domain reflect the psychosocial circumstances, environmental conditions, and the health care system, using a Likert scale ranging from 1 (very poor) to 5 (very good). The SDH-Q demonstrated adequate internal consistency, with Cronbach’s alpha values of 0.917 and 0.939 for structural and intermediary determinants, respectively. Test–retest reliability was also high, with ICC values of 0.938 and 0.941 for structural and intermediary determinants, respectively.

### Data collection

The online Google Survey Form link was employed for data collection by distributing it to the participants who met the study’s inclusion criteria. Google Survey Forms are commonly employed for data collection because they are cost-effective, fast, and easy to access and help reduce missing values [26]. The study inclusion criteria included those from the College of Medicine and Allied Medical Sciences at FUD or the Health Campus of Universiti Sains Malaysia, undergraduate students from first to final year, those registered during the data collection period, and those who consented to participate. We specifically recruited students from medical and health sciences disciplines, consistent with the methodology of the original SDH-Q study, due to their familiarity with the concepts and constructs assessed by the instrument [21]. Participants with prior knowledge of the domains being measured are more likely to understand the items accurately and provide meaningful responses, thereby strengthening the construct validity of the scale [27]. Nevertheless, the SDH-Q is not restricted to this population and has the potential for application among the wider community.

### Content validity

The content validity of the SDH-Q was previously established among Nigerian experts, with Item Content Validity Index (I-CVI) and Scale Content Validity Index (S-CVI) values exceeding the recommended threshold of 0.83 [21]. In the present study, content validity was further assessed among six Malaysian experts from health psychology, public health, and questionnaire development fields. The experts evaluated the relevance of each item to its respective domain using a four-point rating scale (not relevant, somewhat relevant, quite relevant, and highly relevant). I-CVI and S-CVI were calculated following established guidelines [28–30].

### Face validity

In addition, the face validity of the SDH-Q was previously established among Nigerian students, with the Item Face Validity Index (I-FVI) and Scale Face Validity Index (S-FVI) values exceeding the recommended threshold of 0.83 [21]. In the present study, face validity was further assessed among 10 Malaysian students to establish the items’ clarity and comprehension. The students evaluated the clarity and understandability of each item in its respective domain using a four-point rating scale (not clear and understandable, somewhat clear and understandable, clear and understandable, and very clear and understandable). I-FVI and S-FVI were calculated following established guidelines [31,32].

### Sample size

To estimate the sample size for testing the measurement and structural invariance of the SDH-Q, we calculate the sample size based on confirmatory factor analysis (CFA). For CFA testing of seven or fewer constructs, a minimum of 300 participants is recommended [33]. Accordingly, we set 300 as the target CFA sample size in the present study. To account for an anticipated 30% rate of missing data, the sample size was adjusted using the formula 300/(1 − 0.30), resulting in a final adjusted sample of 430 participants per group. Therefore, the total sample consisted of 860 undergraduate students, with 430 from Malaysia and 430 from Nigeria.

### Ethics approval and consent to participate

The Universiti Sains Malaysia’s Human Research Ethics Committee [USM/JEPeM/22110695] granted ethical approval for the study. The participants were informed about the research aim and methods before signing the informed consent form. The investigation conforms to the principles outlined in the Declaration of Helsinki.

### Data analysis

Confirmatory factor analysis (CFA) was performed using Mplus version 8.0 [34] to test the construct validity of the initial hypothesized SDH-Q model. The dataset multivariate normality of the data was investigated using Mardia’s multivariate skewness and kurtosis tests. The tests of skewness (p < .001) and kurtosis (p < .001) reveal a normal distribution of the sample data, demonstrating that the assumption of multivariate normality was not met. Therefore, the robust maximum likelihood estimator (MLR) was applied during the CFA because of its robustness for handling non-normal data distributions and providing accurate parameter estimates even when normality assumptions are violated [34]. A minimum factor loading criterion of 0.40 was applied [35].

The recommended fit indices for a sample size greater than 250 and a number of items between 12 and 30 were a root mean square error of approximation (RMSEA) below 0.07, a standardized root mean square residual (SRMR) below 0.08, and a comparative fit index (CFI) or Tucker–Lewis index (TLI) of 0.92 or higher [36]. The construct validity of the SDH-Q was also investigated using composite reliability (CR) and average variance extracted (AVE). CR was determined using Raykov’s method [37] in Mplus 8.0, with acceptable thresholds of ≥ 0.60 and ≥ 0.50 for CR and AVE, respectively [38,39]. Discriminant validity was established when the factor correlations were below 0.85 [40] and when each factor’s AVE exceeded the squared correlation between the constructs [38]. Finally, model re-specification was conducted by adding residual covariances between items within the same factor based on the modification index (MI) values and when there is theoretical justification.

To establish the measurement and structural invariance of the SDH-Q across the two countries (Malaysia and Nigeria), sequential hierarchical tests of invariance were conducted according to recommended guidelines for assessing measurement invariance [34,41,42]. The SDH-Q model parameters were progressively constrained, and changes in model fit indices were investigated at each step of invariance testing. First, the configural invariance model, which involved fitting a baseline model with no equality constraints across countries, was established and evaluated. Establishing the configural invariance illustrates that the overall factor structure was comparable between groups.

Secondly, the weak or metric invariance model was specified and evaluated. In this model, equality constraints were applied to the factor loadings across the samples of Nigerian and Malaysian students to ensure consistency in the measurement scale and enable precise comparisons between them. Thirdly, the strong invariance model was specified and assessed. In this model, we imposed equality constraints on both factor loadings and item intercepts across the samples of Nigerian and Malaysian students to ensure the comparability of scale factors between them. Finally, the strict invariance model was specified and evaluated. This model applied equality constraints to factor loadings, item intercepts, and residual variances to confirm that the items’ variance in regression equations remained consistent across the two samples of Nigerian and Malaysian students.

The structural invariance of the model parameters was also assessed by evaluating factor variance and covariance invariance, as well as factor means invariance. Factor variance and covariance invariance were tested to determine the similarity of factor correlations between the Nigerian and Malaysian university student samples. Conversely, factor means invariance was examined to identify any differences in factor means across the two groups. Overall, structural invariance analysis aimed to evaluate the extent to which Nigerian students at FUD and Malaysian students at USM differ, regardless of the measurement scale being used.

For this study, we applied the following recommended cut-off values to determine the measurement and structural invariance: an absolute difference (Δ) of 0.01 or less for CFI (ΔCFI) and TLI (ΔTLI), and 0.015 for RMSEA (ΔRMSEA) and (ΔSRMR) [41,43–45]. Furthermore, in this study, we computed the internal consistency of the SDH-Q using Cronbach’s alpha and its stability using test–retest reliability. The intraclass correlation coefficient (ICC) was calculated using a subsample of 70 participants each from Malaysia and Nigeria who completed the questionnaire twice, within a 7-day interval. ICC values above 0.90 were considered excellent stability [46]. The Cronbach’s alpha and ICC were performed using the Statistical Product and Service Solution (SPSS) version 29 (IBM, Armonk, NY, USA).

## Results

### Content validity

All six invited experts from Malaysia provided responses, resulting in a 100% response rate. The I-CVIs ranged from 0.83 to 1, and the S-CVIs were 0.97 and 0.98 for structural determinants of SDH and intermediary determinants of SDH, respectively.

### Face validity

All 10 selected undergraduate students from Malaysia provided responses, resulting in a 100% response rate. The I-FVIs were all equal to 1.00, and the S-FVIs were also equal to 1.00.

### General characteristics of the respondents

Table 1 reveal study participants’ characteristics in the Malaysian sample. There were a total of 430 students (37.4% male, 62.6% female), with a mean age of 21.4 (SD = 1.47). About half of the students were Malay (54.7%) and studying health sciences (45.3%). Additionally, the highest proportion of the students were in Year 2 (52.1%).

**Table 1:** General Characteristics of Malaysian Participants.

Variables	Mean (SD)	n (%)
Age	21.4 (1.47)	
Gender		
Male		161 (37.4)
Female		269 (62.6)
Ethnicity		
Malay		235 (54.7)
Chinese		110 (25.6)
Indian		55 (12.8)
Others		30 (7.0)
Field of study		
Medical sciences		186 (43.3)
Health sciences		195 (45.3)
Dental sciences		49 (11.4)
Study year		
Year 1		95 (22.1)
Year 2		224 (52.1)
Year 3		59 (13.7)
Year 4		43 (10.0)
Year 5		9 (2.1)

SD = standard deviation.

Table 2 reveal study participants’ characteristics in the Nigerian sample. There were a total of 430 students (male 53.0%, female 47.0%), with a mean age of 21.07 (SD = 1.39). More than half of the students were Hausa (68.8%) and studied medicine (52.3%). Additionally, most of the students were in Year 3 (67.2%).

**Table 2:** General Characteristics of Nigeria Participants.

Variables	Mean (SD)	n (%)
Age	21.07 (1.39)	
Gender		
Male		228 (53.0)
Female		202 (47.0)
Ethnicity		
Hausa		296 (68.8)
Yoruba		45 (10.5)
Igbo		15 (3.5)
Others		74 (17.2)
Field of study		
Medicine		225 (52.3)
Human anatomy		117 (27.2)
Human physiology		88 (20.5)
Study year		
Year 1		28 (6.5)
Year 2		14 (3.3)
Year 3		289 (67.2)
Year 4		99 (23.0)

SD = standard deviation.

### Factorial validity of the SDH-Q

**Fig 1:**
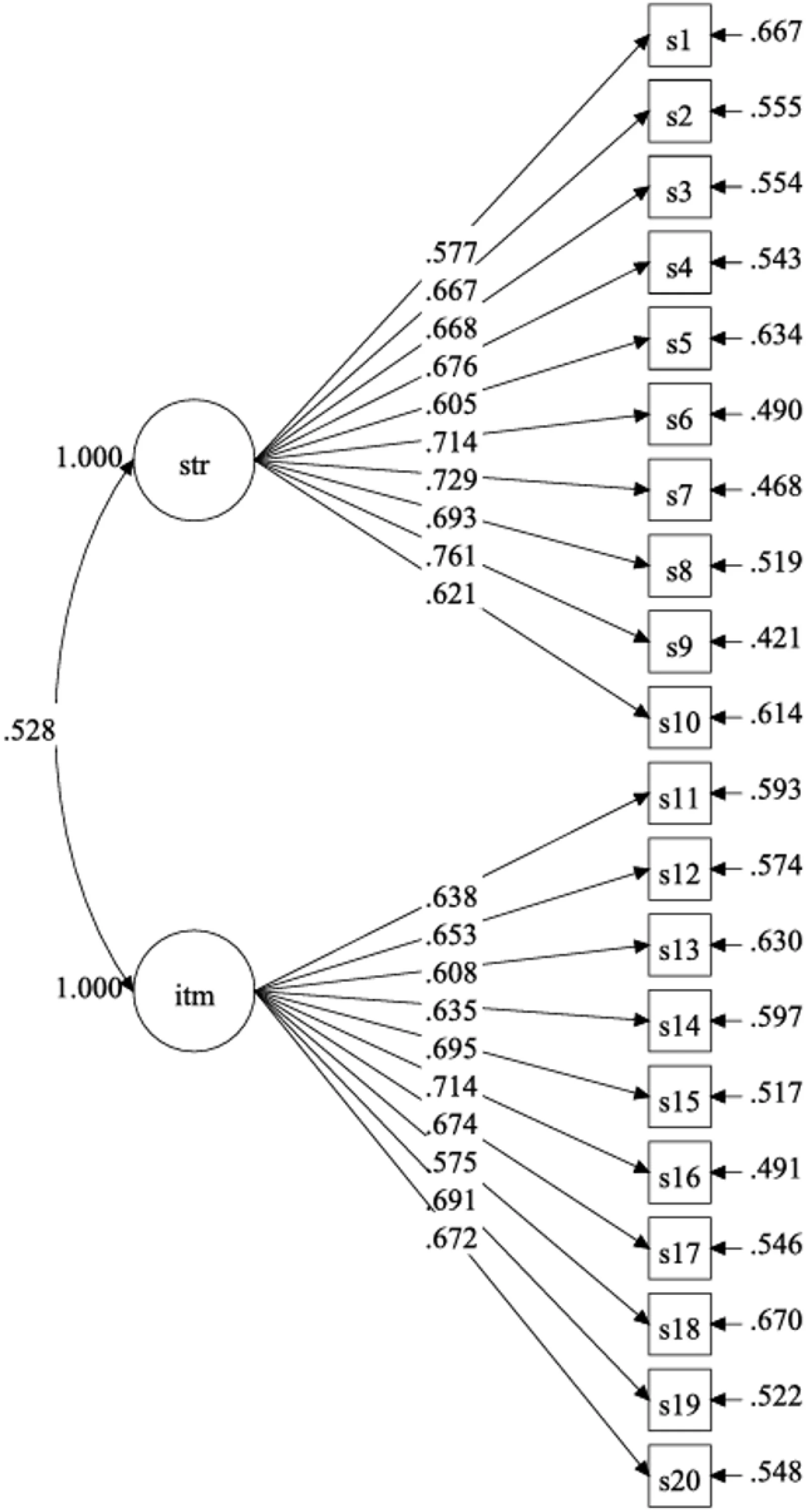
SDH-Q measurement model (Model-1).

**Fig 2:**
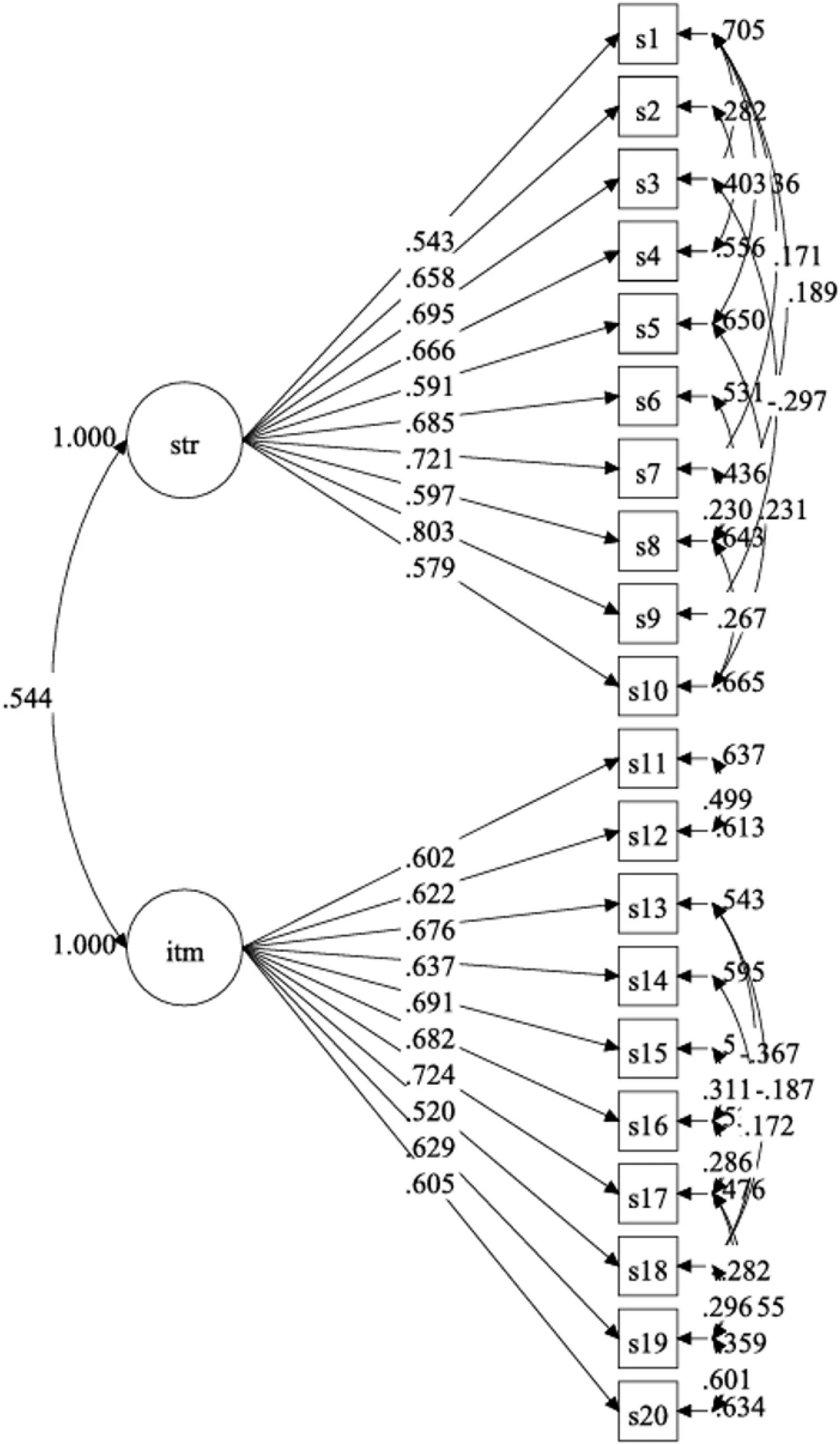
SDH-Q measurement model (Model-2).

The SDH-Q was hypothesized to capture two domains and 20 items (10 items under each domain). The fit indices of the first model (Model 1) were not satisfactory, as indicated in Table 3. However, all of the factor loadings were greater than 0.50 (Fig 1). The fit indices of the Model 1 were adjusted by adding 21 correlated items residuals within the same domain based on the modification indices (see Table 3 and Fig 2). The findings (Model 2) demonstrated satisfactory fit indices (see Table 1). Hence, Model 2 was considered to possess adequate fit indices based on the recommended guidelines, with the factor loadings ranging from 0.520 to 0.803, and all items retained (Fig 2).

**Table 3:** Summary of the SDH-Q model fit indices.

Path model	RMSEA (90% CI)	CFI	TLI	SRMR	RMSEA p-value
Model-1	0.097 (0.092, 0.101)	0.753	0.722	0.068	<.001
Model-2	0.053 (0.048, 0.058)	0.936	0.918	0.044	0.182

Notes: RMSEA = Root Mean Square Error of Approximation, CI = Confidence Interval, CFI = Comparative Fit Index, TLI = Tucker-Lewis Index, SRMR = Standardised Root Mean square Residual, Model-2 with 21 correlated items residual: S20 with S19; S12 with S11; S4 with S2; S8 with S6; S3 with S1; S10 with S8; S16 with S15; S17 with S16; S18 with S13; S10 with S5; S17 with S13; S8 with S7; S9 with S3; S20 with S18; S19 with S18; S19 with S17; S8 with S1; S7 with S1; S18 with S14; S5 with S1; S20 with S17.

### Discriminant and convergent validity

The CR of the final SDH-Q model (Model 2) were 0.839 (structural determinants) and 0.842 (intermediary determinants). The AVE values were 0.433 (structural determinants) and 0.411 (intermediary determinants). The two constructs had a correlation coefficient of 0.182, which is lower than the required threshold of 0.85 for discriminant validity. Furthermore, the squared value of the correlations is less than the AVE values of the two domains, showing an acceptable level of discriminant validity. Table 4 presents the final SDH-Q model’s convergent and discriminant validity based on CR, AVE, correlation coefficient.

**Table 4:** Composite reliability (CR), average variance extraction (AVE), factor correlation and squared correlation for SDH-Q final model.

Domain	CR	AVE	1	2	r^2^
Structural	0.839 (0.816, 0.862)	0.433	1	0.182**	0.033
Intermediary	0.842 (0.809, 0.868)	0.411		1	

Correlation is significant at the 0.001 level (two tailed), r^2^ = squared correlation coefficient.

### Measurement model of the SDH-Q across Malaysia and Nigeria samples

**Fig 3:**
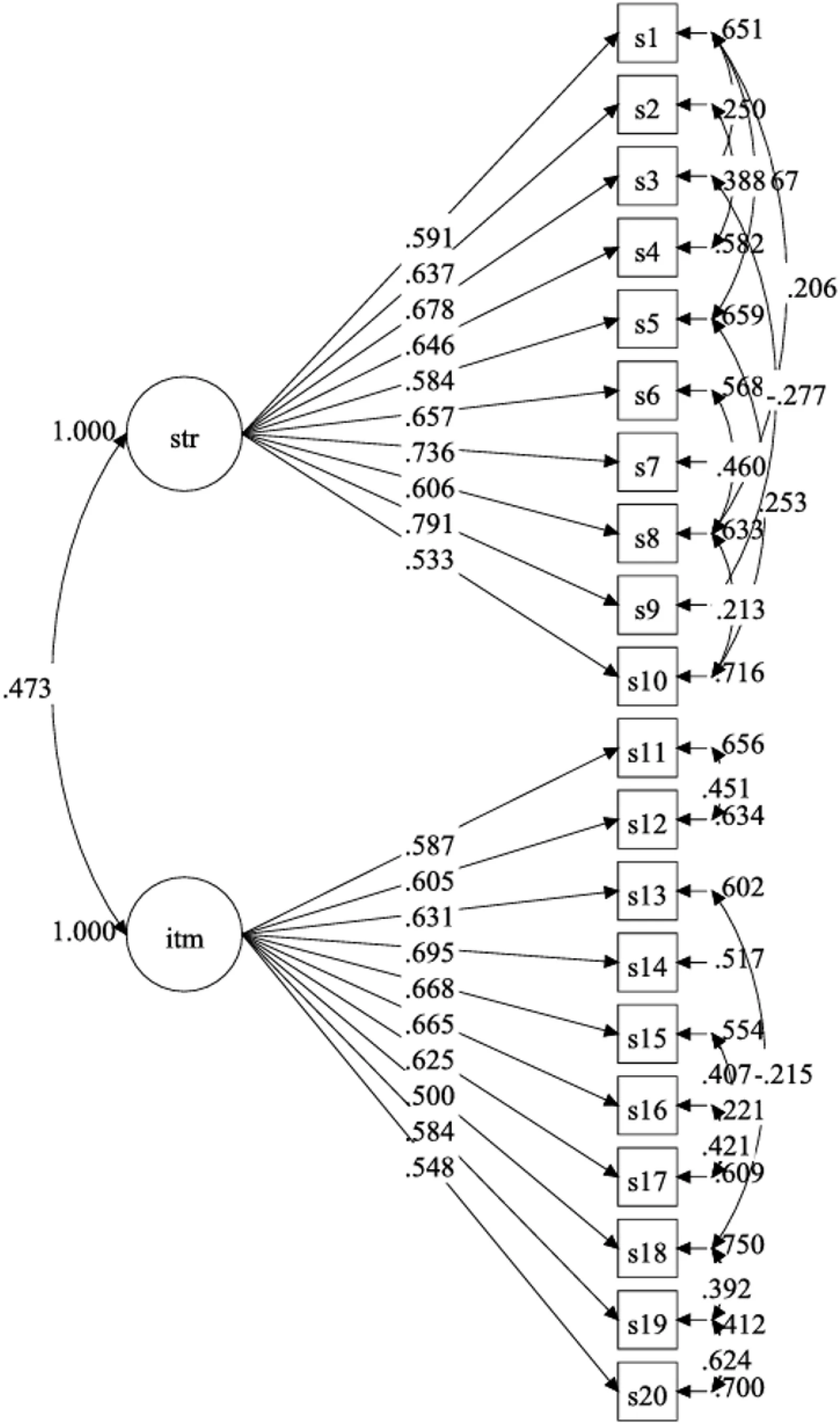
Malaysian SDH-Q measurement model (Model-4).

**Fig 4:**
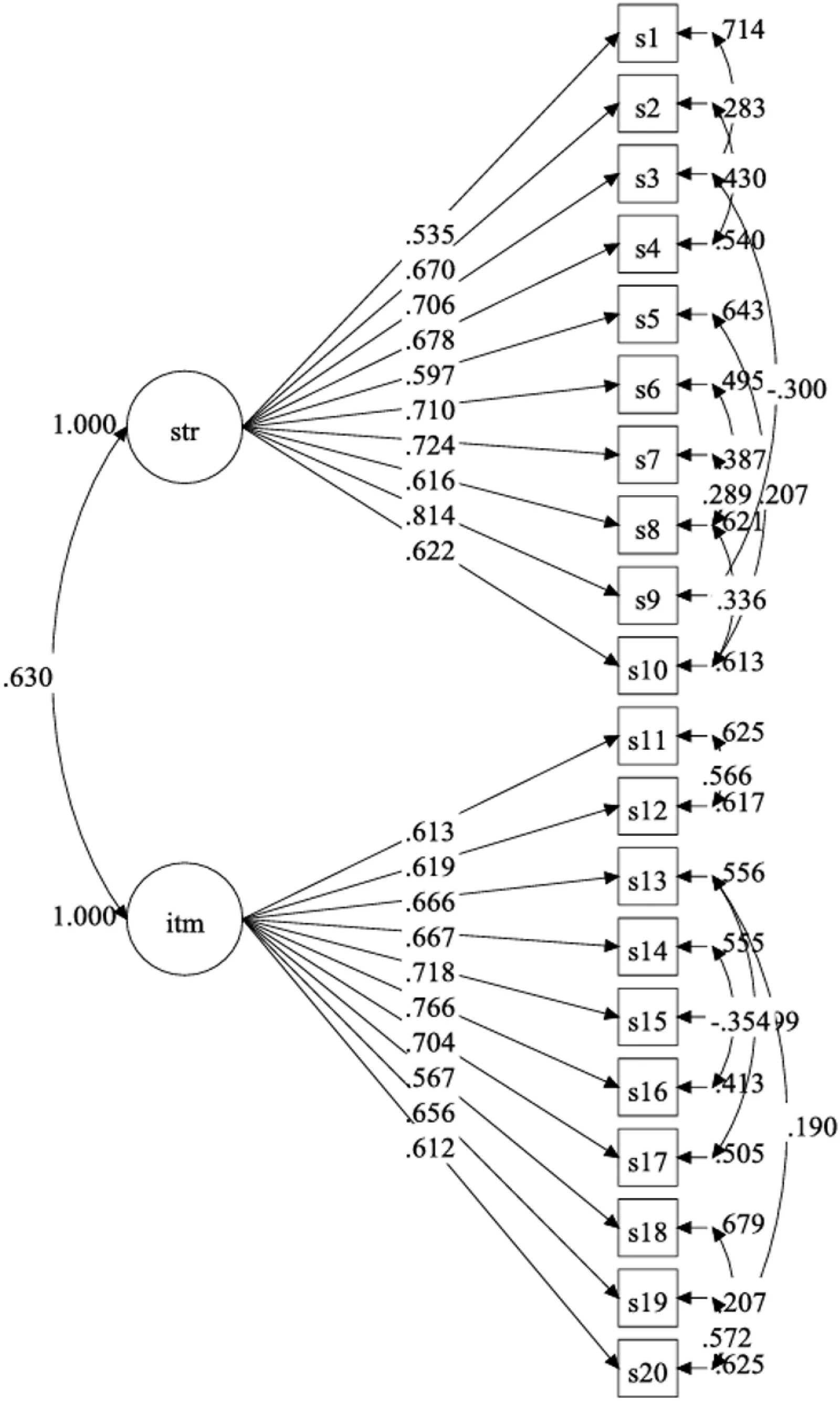
Nigerian SDH-Q measurement model (Model-6).

After the construct validity of the measurement model was determined using the combined samples of Malaysian and Nigerian students, the construct validity of the baseline measurement models for both Malaysia (Model 3) and Nigeria (Model 5) was investigated. These two models did not yield satisfactory fit indices (Table 5). The fit indices were improved after re-specification for both the Malaysian model (RMSEA = 0.055, CFI = 0.928, TLI = 0.910, SRMR = 0.051) and the Nigerian model (RMSEA = 0.052, CFI = 0.935, TLI = 0.921, SRMR = 0.048). The results reveal that the RMSEA, CFI, TLI, and SRMR values were a little different. The final Malaysian model had 16 additional residual covariances (S20 with S19, S12 with S11, S8 with S6, S4 with S2, S3 with S1, S17 with S16, S16 with S15, S18 with S13, S17 with S15, S10 with S5, S20 with S18, S19 with S18, S10 with S8, S8 with S1, S5 with S1, S9 with S3), while the final Nigerian model had 13 added residual covariances (S20 with S19, S12 with S11, S4 with S2, S3 with S1, S8 with S6, S10 with S8, S8 with S7, S17 with S13, S16 with S14, S9 with S3, S10 with S5, S20 with S18, S20 with S13). The resulting factor loadings for Malaysian students and Nigerian students were 0.500 to 0.791 (Fig 3) and 0.535 to 0.814 (Fig 4), respectively.

**Table 5:** Measurement and structural invariance of the SDH-Q.

Models	CFI	TLI	RMSEA	SRMR	Model comparison	ΔCFI	ΔTLI	ΔRMSEA
Model-3 (Malaysian sample: hypothesized)	0.737	0.705	0.099	0.075	–	–	–	–
Model-4^a^ (Malaysian sample: re-specified)	0.928	0.910	0.055	0.051	–	–	–	–
Model-5 (Nigerian sample: hypothesized)	0.779	0.752	0.093	0.067	–	–	–	–
Model-6^b^ (Nigerian sample: re-specified)	0.935	0.921	0.052	0.048	–	–	–	–
Configural (Model-7)	0.931	0.916	0.053	0.050	–	–	–	–
Measurement invariance								
Weak (Model-8)	0.932	0.921	0.052	0.050	8 versus 7	0.001	0.005	−0.001
Strong (Model-9)	0.932	0.925	0.051	0.051	9 versus 8	0	0.004	−0.001
Strict (Model-10)	0.932	0.929	0.049	0.055	10 versus 9	0	0.004	−0.002
Structural invariance								
Factor variance and factor covariance invariance (model-11)	0.932	0.925	0.050	0.058	11 versus 9	0	0	−0.001
Factor variance, covariance, and factor means invariance (model-12)	0.932	0.926	0.050	0.058	12 versus 11	0	0.001	0

^a^Adding residual covariances between item S20 with S19; S12 with S11; S8 with S6; S4 with S2; S3 with S1; S17 with S16; S16 with S15; S18 with S13; S17 with S15; S10 with S5; S20 with S18; S19 with S18; S10 with S8; S8 with S1; S5 with S1; S9 with S3.

^b^Adding residual covariances between item S20 with S19; S12 with S11; S4 with S2; S3 with S1; S8 with S6; S10 with S8; S8 with S7; S17 with S13; S16 with S14; S9 with S3; S10 with S5; S20 with S18; S20 with S13.

### Measurement and structural invariance of the SDH-Q

The Malaysian and Nigerian samples retained all the items in the final model. Hence, the configural model invariance was established by combining the two hypothesized models with similar factor loadings. The configural invariance model (Model-7) fit indices were satisfactory, with the same fixed and free parameter estimates across the countries (Table 3).

Next, the weak measurement invariance model was tested and produced acceptable fit indices (Table 3). In addition, when compared to the non-restrictive configural model, the weak measurement invariance model revealed acceptable metric invariance across countries (ΔCFI = 0.001, ΔTLI = 0.005, ΔRMSEA = −0.001), indicating that Nigerian and Malaysian students interpreted the items similarly. Third, the strong invariance model, a more restrictive model, was tested. The results (ΔCFI = 0, ΔTLI = 0.004, ΔRMSEA = −0.001) showed acceptable metric invariance, suggesting that the factor loadings and intercepts were invariant across countries. Finally, the strict invariance model, the most restrictive, was tested. The results (ΔCFI = 0, ΔTLI = 0.004, ΔRMSEA = −0.002) indicated adequate metric invariance, implying that the items’ mean scores were invariant across countries.

Finally, the structural invariance of the SDH-Q was assessed using the factor variance and factor covariance invariance, and the factor means invariance (Table 3). The factor variance and factor covariance invariance fit indices were satisfactory (CFI = 0.932, TLI = 0.925, SRMR = 0.058, RMSEA = 0.050): also, its differences with the less restrictive invariance model (strong invariance) are within the acceptable values (ΔCFI = 0, ΔTLI = 0, ΔRMSEA = −0.001). These results demonstrated that the correlation between the two factors of the SDH-Q remained the same across the countries. The factor means invariance fit indices were also within the recommended values (CFI = 0.932, TLI = 0.926, SRMR = 0.058, RMSEA = 0.050), and its differences with the less-restrictive model (factor variance and covariance) are within the recommended values (ΔCFI = 0, ΔTLI = 0.001, ΔRMSEA = 0). This result illustrates that the factor means are invariant across the countries.

### Internal consistency

For the Malaysian student sample, the overall Cronbach’s alpha value was 0.951, 0.943 (for structural determinants), and 0.944 (for intermediary determinants). Furthermore, the item-total correlation was 0.634 to 0.870. For the Nigerian student sample, the overall Cronbach’s alpha value was 0.902, 0.917 (for structural determinants), and 0.939 (for intermediary determinants). Furthermore, the item-total correlation was 0.438 to 0.662.

### Test-retest reliability

A total of 70 participants completed the SDH-Q twice within the interval of 7 days. For the Malaysian student sample, the structural determinants of SDH had a mean score of 37.8 (SD = 4.88) on day 1 and 37.4 (SD = 4.42) on day 7, with an ICC value of 0.780 (95% CI: 0.646, 0.863, p-value < 0.001). The intermediary determinants of SDH had a mean score of 37.4 (SD = 4.42) on day 1 and 37.3 (SD = 4.37) on day 7, with an ICC value of 0.799 (95% CI: 0.677, 0.875, p-value < 0.001). For the Nigerian student sample, the structural determinants of SDH had a mean score of 38.8 (SD = 4.77) on day 1 and 37.4 (SD = 5.53) on day 7, with an ICC value of 0.938 (95% CI: 0.901, 0.961, p-value < 0.001). The intermediary determinants of SDH had a mean score of 37.5 (SD = 5.37) on day 1 and 37.2 (SD = 4.37) on day 7, with an ICC value of 0.941 (95% CI: 0.907, 0.963, p-value < 0.001).

## Discussion

The constructs and items of the SDH-Q were developed based on the conceptual framework for action on the social determinants of health proposed by Solar and Irwin [3]. Within this framework, SDH are classified into two broad domains: structural determinants and intermediary determinants. The items measuring structural determinants of SDH were designed to capture factors that shape social hierarchy and influence individuals’ socioeconomic positions, including income, education, occupation, social class, gender, race or ethnicity, and material conditions [1,3]. These determinants are often shaped by historical, social, economic, and political processes, including public policies. In contrast, the items assessing intermediary determinants of SDH focused on conditions that directly influence health outcomes, including psychosocial circumstances, behavioural and biological factors, and the quality of the healthcare system [1,3].

The SDH comprises the social factors that influence the health of individuals and populations, and the social courses that cause unequal circulations of resources among groups with differing social status [5,9,47]. These SDH factors consisted of financial status, literacy, employment status, social class, sex, ethnicity, material conditions, psychosocial conditions, as well as behavioural and biological or genetic factors, classified broadly into structural and intermediary determinants of health [1,4]. Recently, researchers have increasingly sought to establish and validate a unified measure for evaluating perceived satisfaction with these SDH determinants.

The STBH-Q developed by Oster et al., [18] contains five underlying constructs comprising 16 items. The constructs were family and childhood, employment, finances and education, access, physical and mental health, and safety at home and in the community. However, there was cross-loading of items throughout the study’s EFA process. Consequently, the study by Sabo et al., [21] attempts to resolve these issues by creating a similar scale with two constructs, namely, structural determinants of health and intermediary determinants of health, which is in line with the WHO’s CSDH work [1]. Patton et al., [48] demonstrated that supportive relationships with family, schools, and peers play an important role in helping young people reach their full potential; nonetheless, broader structural conditions, such as national economic status and income, remain the most important determinants of health globally. Therefore, the present study evaluated the measurement and structural invariance of the SDH-Q among university students in Malaysia and Nigeria.

The CFA results of the present study illustrated that the SDH-Q with a 20-item and 2-factor model showed sufficient fit indices in both the Malaysian and Nigerian samples as well as the combined sample, and all the items had acceptable factor loading on their respective constructs (> 0.50). These demonstrate that the SDH scale has acceptable psychometric properties and can be applied to assess individuals’ perceived social determinants of health across Malaysia and Nigeria [38,40,42,45], with the actual factor loadings ranging between 0.500 and 0.814. The present study was the first to test the construct validity of the SDH scale among the Malaysian population; as such, we could not compare the current study with prior findings in Malaysia. However, our study confirms the previous study by Sabo et al., [21], which reported the SDH-Q to possess acceptable psychometric properties, with factor loadings ranging between 0.435 and 0.780.

The findings reveal that the SDH-Q model demonstrated good reliability and acceptable discriminant validity in the combined sample. Composite reliability values were above the recommended 0.6 [39], while AVE values were slightly below the recommended threshold of 0.5 [38]. However, this is acceptable if the CR values are above 0.60 [38]. The correlation between the two domains (0.182) and its squared value were below the corresponding AVEs, supporting discriminant validity. The Cronbach’s alpha was high in both countries, above 0.90, indicating excellent internal consistency reliability [46]. ICC values for Malaysian students were 0.780 (structural) and 0.799 (intermediary), while Nigerian students demonstrated higher stability with ICC values of 0.938 (structural) and 0.941 (intermediary). The SDH-Q in this study showed adequate reliability and stability across both countries. Similarly, the original SDH-Q among Nigerian students shows all Cronbach’s alpha above 0.90, and the CR was 0.797 for structural determinants and 0.794 for intermediary determinants [21].

The measurement invariance of the SDH-Q was investigated across Malaysian and Nigerian university students by applying the configural, weak, strong, and strict invariance models [41,49]. The findings reveal that SDH-Q was invariant across the countries, indicating that both the students possessed similar understandings and interpretations of the SDH-Q constructs and items [41,49]. Establishing the measurement invariance of the SDH-Q is crucial for ensuring the validity and reliability of cross-country comparisons of these social determinants of health factors. Furthermore, the structural invariance of these scales was favourable for factor variance and covariance, and the factor means invariance across the two countries. These findings reveal that the strength of relationships between the factors remains stable, and there was no significant mean difference in total scores for structural and intermediary determinants between the Malaysian and Nigerian university students [41,49]. Hence, the SDH-Q can be used to make valid comparisons across different cultures.

Error covariances were added in the present study to make adjustment in the model’s fit indices. These error covariances reflects common sources of variances outside the constructs in the model [39]. These included error covariances added in this study were 21 (overall model), 16 (Malaysian students), and 13 (Nigerian students). The MI values reported in Mplus were used as a guide for including all the error covariances and with suitable theoretical backing. Previous studies have reported that error covariances can be added when they have theoretical meaning [45,50]. Also, the original SDH-Q had 15 pairs of error covariances between items within the same domain (8 for structural determinants of health and 7 for intermediary determinants of health) [21].

The current study was the first to test the cross-cultural validity of the perceived SDH with a sufficient sample size (100% response rate). However, the study is not without some limitations. The study used a self-reported measure of SDH-Q, which is associated with some information bias and social desirability bias, which could be prevalent among medical students responding to psychosocial constructs, consequently decreasing the accuracy of the data collected. The participants were assured that their information would be kept confidential and encouraged to respond honestly based on their actual perceptions. Additionally, the use of a convenience sampling method in selecting the study participants may limit the generalizability of the findings. The study also did not determine other forms of validity, including concurrent or predictive validity. Future research should therefore incorporate these validity measures as well as employ probability sampling techniques to further test the reliability and validity of the SDH-Q.

## Conclusion

The study determines the psychometric properties of the SDH-Q for evaluating perceived social determinants of health among university students in Malaysian and Nigerian. The SDH-Q was shown to have acceptable convergent and discriminant validity based on various fit indices. Moreover, the SDH-Q revealed acceptable levels of measurement invariance and structural invariance. These results indicate that the SDH-Q can be reliably used to make meaningful comparisons between Malaysian and Nigerian students.

## Supporting information

S1 Dataset Anonymized dataset used for CFA. (SAV)

S1 Questionnaire Social Determinants of Health Questionnaire. (PDF)

## Abbreviations

FUD: Federal University Dutse
SDH: Social Determinants of health
CSDH: Commission on social determinants of health
CFA: Confirmatory factor analysis
CR: Composite reliability
AVE: Average variance extracted
ICC: Intraclass correlation coefficient
