# Supplementary material for: Measurement and structural invariance of the social determinants of health questionnaire (SDH-Q) across countries
Source: PLoS One. 2026 Aug 27;21(8):e0356884. doi: 10.1371/journal.pone.0356884 (PMC13521408; doi:10.1371/journal.pone.0356884)
Supplement: Supplementary file 1 [file pone.0356884.s002.pdf]

SOCIAL DETERMINANTS OF HEALTH QUESTIONNAIRE

Instruction: *This scale consists of 20 items that evaluate perceived social determinants of health. Please rate each item by selecting the appropriate number. There are no right or wrong answers; we are only interested in how you feel about each question.*

1	How satisfied are you with your gender?	1	2	3	4	5
2	How do you rate the state of your current housing or accommodations?	1	2	3	4	5
3	In terms of all the opportunities in your community, how would you evaluate gender equality?	1	2	3	4	5
4	How do you rate the availability of healthy food or safe water in your neighbourhood?	1	2	3	4	5
5	How satisfied are you with your ethnic background?	1	2	3	4	5
6	How do you rate the support you received from your family members?	1	2	3	4	5
7	In terms of all the opportunities in your community, how would you evaluate ethnic equality?	1	2	3	4	5
8	How do you rate the support you received from your friends?	1	2	3	4	5
9	How satisfied are you with your present financial income?	1	2	3	4	5
10	How do you rate the state of your mental health?	1	2	3	4	5
11	How do you rate your financial opportunities in the future?	1	2	3	4	5
12	How do you rate the state of your physical health?	1	2	3	4	5
13	How satisfied are you with your present education?	1	2	3	4	5
14	How would you rate your good lifestyle habits, such as healthy eating?	1	2	3	4	5
15	How do you rate your employment opportunity in the future?	1	2	3	4	5
16	How do you rate the quality of the health system services in your community?	1	2	3	4	5
17	How satisfied are you with your present standard of living?	1	2	3	4	5
18	How do you rate your access to health services when needed?	1	2	3	4	5
19	How do you rate the government's effort towards improving your standard of living?	1	2	3	4	5
20	How do you rate the affordability of health services in your community?	1	2	3	4	5

Note:

Factors and items

1) Structural determinants of social determinants of health: 1, 3, 5, 7, 9, 11, 13, 15, 17, 19 [rating: 1 = totally unsatisfied, 2 = unsatisfied, 3 = somewhat satisfied, 4 = satisfied, 5 = totally satisfied].
2) Intermediary social determinants of health: 2, 4, 6, 8, 10, 12, 14, 16, 18, 20 [rating: 1 = very poor, 2 = poor, 3 = somewhat good, 4 = good, 5 = very good].
