# Supplementary material for: Measurement and structural invariance of the social determinants of health questionnaire (SDH-Q) across countries
Source: PLoS One. 2026 Aug 27;21(8):e0356884. doi: 10.1371/journal.pone.0356884 (PMC13521408; doi:10.1371/journal.pone.0356884)
Supplement: Supplementary file 2 [file pone.0356884.s004.docx]

**Dear Editor, Dr. Taher Hatahet:**

On behalf of my co-authors, I would like to thank you for the opportunity to revise our manuscript entitled **"Measurement and Structural Invariance of the Social Determinants of Health Questionnaire (SDH-Q) across Countries."**

We sincerely appreciate the constructive comments and suggestions provided by the editor and reviewers. We have carefully considered each comment and believe that we have adequately addressed all of the concerns raised. We hope that the revised manuscript is now suitable for publication and will make a valuable contribution to your journal.

Should there be any remaining issues that require our attention, we would be grateful for the opportunity to address them further.

Below, we provide our detailed responses, highlighted in yellow, to the journal requirements and the reviewers' comments.

**Journal requirements:**

1. When submitting your revision, we need you to address these additional requirements.

Please ensure that your manuscript meets PLOS ONE's style requirements, including those for file naming. The PLOS ONE style templates can be found at

https://journals.plos.org/plosone/s/file?id=wjVg/PLOSOne_formatting_sample_main_body.pdf and

<https://journals.plos.org/plosone/s/file?id=ba62/PLOSOne_formatting_sample_title_authors_affiliations.pdf>

Response: Thank you for bringing this to our attention. We have carefully reviewed the manuscript and revised it to comply with the PLOS ONE style requirements. The manuscript formatting, including title page structure, author affiliations, section organization, and file naming, has been checked against the PLOS ONE formatting templates provided and updated where necessary.

2. Thank you for stating in your Funding Statement:

This research was supported by the Universiti Sains Malaysia Short-Term Grant, Grant No. R501-LR-RND002-0000000147-0000.

Please provide an amended statement that declares *all* the funding or sources of support (whether external or internal to your organization) received during this study, as detailed online in our guide for authors at http://journals.plos.org/plosone/s/submit-now.  Please also include the statement “There was no additional external funding received for this study.” in your updated Funding Statement.

Please include your amended Funding Statement within your cover letter. We will change the online submission form on your behalf.

Response: Thank you for your comment. We have revised the Funding Statement to ensure that all sources of support received during this study are fully declared. The amended Funding Statement is provided below and has been included in the cover letter as requested:

"This research was supported by the Universiti Sains Malaysia Short-Term Grant (Grant No. R501-LR-RND002-0000000147-0000). There was no additional external funding received for this study."

3. Thank you for stating the following financial disclosure:

This research was supported by the Universiti Sains Malaysia Short-Term Grant, Grant No. R501-LR-RND002-0000000147-0000.

Please state what role the funders took in the study.  If the funders had no role, please state: "The funders had no role in study design, data collection and analysis, decision to publish, or preparation of the manuscript."

If this statement is not correct you must amend it as needed.

Please include this amended Role of Funder statement in your cover letter; we will change the online submission form on your behalf.

Response: Thank you for your comment. We have updated the Role of Funder statement and included it in the cover letter as requested.

**Role of Funder Statement:**

*This research was supported by the Universiti Sains Malaysia Short-Term Grant (Grant No. R501-LR-RND002-0000000147-0000). The funders had no role in study design, data collection and analysis, decision to publish, or preparation of the manuscript.*

4. Your ethics statement should only appear in the Methods section of your manuscript. If your ethics statement is written in any section besides the Methods, please move it to the Methods section and delete it from any other section. Please ensure that your ethics statement is included in your manuscript, as the ethics statement entered into the online submission form will not be published alongside your manuscript.

Response: Thank you for your comment. We have reviewed the manuscript and confirmed that the ethics statement is included in the Methods section. Any duplicate ethics statements appearing elsewhere in the manuscript have been removed to comply with PLOS ONE requirements.

5. We note that there is identifying data in the Supporting Information file <SDH_invariance.sav>. Due to the inclusion of these potentially identifying data, we have removed this file from your file inventory. Prior to sharing human research participant data, authors should consult with an ethics committee to ensure data are shared in accordance with participant consent and all applicable local laws.

Data sharing should never compromise participant privacy. It is therefore not appropriate to publicly share personally identifiable data on human research participants. The following are examples of data that should not be shared:

-Name, initials, physical address

-Ages more specific than whole numbers

-Internet protocol (IP) address

-Specific dates (birth dates, death dates, examination dates, etc.)

-Contact information such as phone number or email address

-Location data

-ID numbers that seem specific (long numbers, include initials, titled “Hospital ID”) rather than random (small numbers in numerical order)

Data that are not directly identifying may also be inappropriate to share, as in combination they can become identifying. For example, data collected from a small group of participants, vulnerable populations, or private groups should not be shared if they involve indirect identifiers (such as sex, ethnicity, location, etc.) that may risk the identification of study participants.

Additional guidance on preparing raw data for publication can be found in our Data Policy (https://journals.plos.org/plosone/s/data-availability#loc-human-research-participant-data-and-other-sensitive-data) and in the following article: http://www.bmj.com/content/340/bmj.c181.long.

Please remove or anonymize all personal information (<specific identifying information in file to be removed>), ensure that the data shared are in accordance with participant consent, and re-upload a fully anonymized data set. Please note that spreadsheet columns with personal information must be removed and not hidden as all hidden columns will appear in the published file.

Response: Thank you for your comment. We reviewed the Supporting Information file (SDH_invariance.sav) and identified two variables (participant ID and country) that could potentially contribute to participant identification. These variables have been removed from the dataset. The revised dataset now contains only the questionnaire variables used in the confirmatory factor analysis (CFA) and does not include any direct personal identifiers. We have ensured that the shared data are fully anonymized and comply with participant consent requirements and PLOS ONE's Data Availability Policy. A revised anonymized dataset has been re-uploaded (dataset.sav).

6. Please include captions for your Supporting Information files at the end of your manuscript, and update any in-text citations to match accordingly. Please see our Supporting Information guidelines for more information: <http://journals.plos.org/plosone/s/supporting-information>.

Response: Thank you for your comment. We have added captions for all Supporting Information files at the end of the manuscript in accordance with the PLOS ONE Supporting Information guidelines. We have also reviewed and updated the corresponding in-text citations to ensure consistency with the Supporting Information file designations.

7. If the reviewer comments include a recommendation to cite specific previously published works, please review and evaluate these publications to determine whether they are relevant and should be cited. There is no requirement to cite these works unless the editor has indicated otherwise.

Response: We reviewed all reviewer comments and noted that no specific publications were recommended for citation. Nevertheless, we carefully evaluated the literature cited throughout the manuscript and updated relevant references where appropriate to support the revised content.

8. Please review your reference list to ensure that it is complete and correct. If you have cited papers that have been retracted, please include the rationale for doing so in the manuscript text, or remove these references and replace them with relevant current references. Any changes to the reference list should be mentioned in the rebuttal letter that accompanies your revised manuscript. If you need to cite a retracted article, indicate the article’s retracted status in the References list and also include a citation and full reference for the retraction notice.

Response: The reference list has been reviewed for completeness and accuracy. No retracted references were identified, and all references were confirmed to be relevant and current.

**Editor comments:**

thanks for submitting your article to PLOS ONE. Reviewers comments are provided below. Please address them with mainly the addition of theoretical formwork for the study questionnaire to be expanded in the introduction.

Response: Thank you for your valuable comments and for the reviewers' constructive feedback. In response to your recommendation, we have substantially expanded the theoretical framework underpinning the Social Determinants of Health Questionnaire (SDH-Q) in the Introduction section. Specifically, we provided a more detailed description of the Commission on Social Determinants of Health (CSDH) framework, the conceptual basis of the structural and intermediary determinants of health, and the rationale for how these constructs are operationalized in the SDH-Q. We also strengthened the justification for examining measurement and structural invariance across Malaysia and Nigeria. In addition, we carefully addressed all reviewer comments and have detailed our responses in the accompanying rebuttal letter. We appreciate the opportunity to revise our manuscript and believe that these revisions have strengthened the conceptual foundation and overall quality of the study.

**Reviewer 1:**

1. The research is written in grammatically and scientifically correct language. The statistical analyses were accurate. It would have been preferable for the researcher to include a copy of the questionnaire as an appendix at the end of the research.

Response: We appreciate the reviewers’ comments and suggestions. We have included the questionnaire as a supporting information file during the submission process, in accordance with the journal’s requirements.

2. The exploratory factor analysis should have been performed before the confirmatory factor analysis.

Response: We thank the reviewer for this valuable comment. Exploratory Factor Analysis (EFA) was not conducted in the present study because the SDH-Q had already been developed and validated by previous authors, who established its factor structure through both EFA and Confirmatory Factor Analysis (CFA). The primary objective of this study was to evaluate the measurement and structural invariance of the SDH-Q across Nigeria and Malaysia rather than to re-explore its underlying factor structure. Also, we have added results of content and face validity in lines 163 – 179, and 253 – 259.

3. The scientific references should have been arranged in accordance with APA7.

Response: We thank the reviewer for this suggestion. However, the references in the present study were done in accordance with the journal's requirement, which is the Vancouver reference style.

4. The discussion of the questionnaire appears to have been insufficient in terms of the theoretical framework underpinning its design.

Response: We thank the reviewer for this valuable comment. We have now added more information on the theoretical development of the SDH-Q in lines 395 - 405.

**Reviewer 2:**

This manuscript tilted "Measurement and Structural Invariance of the Social Determinants of Health Questionnaire (SDH-Q) across Countries" is a cross-sectional study that explores the psychometric properties of the SDH-Q among 860 undergraduate medical students from Malaysia and Nigeria. While the topic is commendable and timely, several core psychometric and methodological concerns must be addressed to strengthen the validity of the findings.

1. The comparative approach between Malaysia and Nigeria is intriguing. However, the manuscript would benefit from a more robust theoretical rationale explaining the selection of these specific populations (for example, are there national indicators that underscore the practical importance?).

Response: We thank the reviewer for this valuable comment. We have now added more information on the theoretical rationale for testing the measurement and structural invariance of the SDH-Q across Nigeria and Malaysia in our study (lines 94 – 117).

2. The authors must clarify the primary scope of this study: Is the objective to validate an instrument specifically for a medical student population, or is it intended for broader community settings to measure SDH? This is crucial in highlighting the importance of the research and in determining a concise research question.

Response: We thank the reviewer for this valuable comment. We have now added more information on the primary scope of the SDH-Q in lines 155 – 161.

3. The absence of a Pilot Study to establish Face Validity is a procedural gap. In cross-cultural psychometrics, pre-testing is essential to ensure that items are interpreted consistently across different linguistic and cultural landscapes.

Response: We thank the reviewer for this valuable comment. In the present study, we assessed the content and face validity of the SDH-Q among Malaysian experts and students, respectively, as these validity assessments had already been conducted and reported in the original SDH-Q development study involving Nigerian experts and students. The results were initially omitted to maintain a concise manuscript. However, we have now added the findings from the content and face validity assessments conducted in the current study and have also provided the corresponding results from the original Nigerian validation study for comparison and completeness (lines 163 – 179, and 253 – 259).

4. The integrity of statistical outcomes is inseparable from the quality of the data inputs. Moving directly to a full-scale analysis without a pilot study introduces a high risk of measurement error. Thus, if the items were misinterpreted by participants, the resulting statistical models will be mathematically flawed regardless of their complexity.

Response: We thank the reviewer for this valuable comment. Exploratory Factor Analysis (EFA) was not conducted in the present study because the SDH-Q had already been developed and validated by previous authors, who established its factor structure through both EFA and Confirmatory Factor Analysis (CFA). The primary objective of this study was to evaluate the measurement and structural invariance of the SDH-Q across Nigeria and Malaysia rather than to re-explore its underlying factor structure. Also, we have added results of content and face validity in lines 163 – 179 and 253 – 259.

5. The authors acknowledge information bias but there is a need to specifically address social desirability bias, which could be prevalent among medical students responding to psychosocial constructs.

Response: We thank the reviewer for this valuable comment. We have now included this in the study limitation in lines 473 – 476.

6. The manuscript's narrative is generally clear, but the introduction requires better thematic alignment. Specifically, the information regarding Sustainable Development Goals (SDGs) should not be presented as general background; instead, it must be strategically linked to the scale’s constructs and its field application. For instance, the authors should condense the broad descriptions of SDGs to focus exclusively on those most relevant to the study (e.g., SDG 3: Good Health and Well-being and SDG 10: Reduced Inequalities). This would allow more space to discuss the practical significance of the scale, specifically how its results can inform field-level interventions and evidence-based policymaking in Malaysia and Nigeria.

Response: We thank the reviewer for this valuable comment. We have made amendments to the introduction as suggested by the reviewer in lines 94 – 117.

7. Moreover, there is a logical inconsistency in the introduction’s rationale. The authors justify the need for this study by citing Sabo et al. (2024) (P 5, paragraph 2, line 1; information cited from the reference [27]); however, the data collection for the current study was undertaken in 2023 (P 5, study design ( paragraph 1, line 5)). The authors should clarify this timeline or provide the original sources that informed the study's inception.

Response: Response: We thank the reviewer for this valuable comment. We have revised and corrected the data collection period in the manuscript. The previously reported dates were included due to an oversight, as the authors had referred to information from their study published in 2024. The updated data collection dates have now been accurately reflected and supported by the ethical approval extension letter, which has been provided as a supplementary file.
