# Supplementary material for: Measurement and structural invariance of the social determinants of health questionnaire (SDH-Q) across countries
Source: PLoS One. 2026 Aug 27;21(8):e0356884. doi: 10.1371/journal.pone.0356884 (PMC13521408; doi:10.1371/journal.pone.0356884)
Supplement: Peer review history

# Peer review: Decision Letter 0

**DOI:** 10.1371/journal.pone.0356884.r001

**Authors:** Taher Hatahet

3 Jun 2026

Dear Dr. Kueh,

Thank you for submitting your manuscript to PLOS ONE. After careful consideration, we feel that it has merit but does not fully meet PLOS ONE’s publication criteria as it currently stands. Therefore, we invite you to submit a revised version of the manuscript that addresses the points raised during the review process.

Please submit your revised manuscript by Jul 18 2026 11:59PM. If you will need more time than this to complete your revisions, please reply to this message or contact the journal office at plosone@plos.org. When you're ready to submit your revision, log on to https://www.editorialmanager.com/pone/ and select the 'Submissions Needing Revision' folder to locate your manuscript file.

- A letter that responds to each point raised by the academic editor and reviewer(s). You should upload this letter as a separate file labeled 'Response to Reviewers'.
- A marked-up copy of your manuscript that highlights changes made to the original version. You should upload this as a separate file labeled 'Revised Manuscript with Track Changes'.
- An unmarked version of your revised paper without tracked changes. You should upload this as a separate file labeled 'Manuscript'.

If you would like to make changes to your financial disclosure, please include your updated statement in your cover letter. Guidelines for resubmitting your figure files are available below the reviewer comments at the end of this letter.

If applicable, we recommend that you deposit your laboratory protocols in protocols.io to enhance the reproducibility of your results. Protocols.io assigns your protocol its own identifier (DOI) so that it can be cited independently in the future. For instructions see: https://journals.plos.org/plosone/s/submission-guidelines#loc-laboratory-protocols. Additionally, PLOS ONE offers an option for publishing peer-reviewed Lab Protocol articles, which describe protocols hosted on protocols.io. Read more information on sharing protocols at https://plos.org/protocols?utm_medium=editorial-email&utm_source=authorletters&utm_campaign=protocols.

As the corresponding author, your ORCID iD is verified in the submission system and will appear in the published article. PLOS supports the use of ORCID, and we encourage all coauthors to register for an ORCID iD and use it as well. Please encourage your coauthors to verify their ORCID iD within the submission system before final acceptance, as unverified ORCID iDs will not appear in the published article. *Only*  the individual author can complete the verification step; PLOS staff *cannot*  verify ORCID iDs on behalf of authors.

We look forward to receiving your revised manuscript.

Kind regards,

Taher Hatahet, Ph.D

Academic Editor

PLOS One

**Journal Requirements:**

1. When submitting your revision, we need you to address these additional requirements.

Please ensure that your manuscript meets PLOS ONE's style requirements, including those for file naming. The PLOS ONE style templates can be found at

https://journals.plos.org/plosone/s/file?id=wjVg/PLOSOne_formatting_sample_main_body.pdf and

https://journals.plos.org/plosone/s/file?id=ba62/PLOSOne_formatting_sample_title_authors_affiliations.pdf

2. Thank you for stating in your Funding Statement:

This research was supported by the Universiti Sains Malaysia Short-Term Grant, Grant No. R501-LR-RND002-0000000147-0000.

Please provide an amended statement that declares *all* the funding or sources of support (whether external or internal to your organization) received during this study, as detailed online in our guide for authors at http://journals.plos.org/plosone/s/submit-now.  Please also include the statement “There was no additional external funding received for this study.” in your updated Funding Statement.

Please include your amended Funding Statement within your cover letter. We will change the online submission form on your behalf.

3. Thank you for stating the following financial disclosure:

This research was supported by the Universiti Sains Malaysia Short-Term Grant, Grant No. R501-LR-RND002-0000000147-0000.

Please state what role the funders took in the study.  If the funders had no role, please state: "The funders had no role in study design, data collection and analysis, decision to publish, or preparation of the manuscript."

If this statement is not correct you must amend it as needed.

Please include this amended Role of Funder statement in your cover letter; we will change the online submission form on your behalf.

4. Your ethics statement should only appear in the Methods section of your manuscript. If your ethics statement is written in any section besides the Methods, please move it to the Methods section and delete it from any other section. Please ensure that your ethics statement is included in your manuscript, as the ethics statement entered into the online submission form will not be published alongside your manuscript.

5. We note that there is identifying data in the Supporting Information file <SDH_invariance.sav>. Due to the inclusion of these potentially identifying data, we have removed this file from your file inventory. Prior to sharing human research participant data, authors should consult with an ethics committee to ensure data are shared in accordance with participant consent and all applicable local laws.

Data sharing should never compromise participant privacy. It is therefore not appropriate to publicly share personally identifiable data on human research participants. The following are examples of data that should not be shared:

-Name, initials, physical address

-Ages more specific than whole numbers

-Internet protocol (IP) address

-Specific dates (birth dates, death dates, examination dates, etc.)

-Contact information such as phone number or email address

-Location data

-ID numbers that seem specific (long numbers, include initials, titled “Hospital ID”) rather than random (small numbers in numerical order)

Data that are not directly identifying may also be inappropriate to share, as in combination they can become identifying. For example, data collected from a small group of participants, vulnerable populations, or private groups should not be shared if they involve indirect identifiers (such as sex, ethnicity, location, etc.) that may risk the identification of study participants.

Additional guidance on preparing raw data for publication can be found in our Data Policy (https://journals.plos.org/plosone/s/data-availability#loc-human-research-participant-data-and-other-sensitive-data) and in the following article: http://www.bmj.com/content/340/bmj.c181.long.

Please remove or anonymize all personal information (<specific identifying information in file to be removed>), ensure that the data shared are in accordance with participant consent, and re-upload a fully anonymized data set. Please note that spreadsheet columns with personal information must be removed and not hidden as all hidden columns will appear in the published file.

6. Please include captions for your Supporting Information files at the end of your manuscript, and update any in-text citations to match accordingly. Please see our Supporting Information guidelines for more information: http://journals.plos.org/plosone/s/supporting-information.

7. If the reviewer comments include a recommendation to cite specific previously published works, please review and evaluate these publications to determine whether they are relevant and should be cited. There is no requirement to cite these works unless the editor has indicated otherwise. 

8. Please review your reference list to ensure that it is complete and correct. If you have cited papers that have been retracted, please include the rationale for doing so in the manuscript text, or remove these references and replace them with relevant current references. Any changes to the reference list should be mentioned in the rebuttal letter that accompanies your revised manuscript. If you need to cite a retracted article, indicate the article’s retracted status in the References list and also include a citation and full reference for the retraction notice.

**Additional Editor Comments:**

Dear Author

thanks for submitting your article to PLOS ONE. Reviewers comments are provided below.

Please address them with mainly the addition of theoretical formwork for the study questionnaire to be expanded in the introduction.

Kind regards

Reviewers' comments:

**Comments to the Author**

1. Is the manuscript technically sound, and do the data support the conclusions?

Reviewer #1: Yes

Reviewer #2: Partly

**********

2. Has the statistical analysis been performed appropriately and rigorously?

Reviewer #1: Yes

Reviewer #2: No

**********

3. Have the authors made all data underlying the findings in their manuscript fully available?

The PLOS Data policy

Reviewer #1: Yes

Reviewer #2: Yes

**********

4. Is the manuscript presented in an intelligible fashion and written in standard English?

Reviewer #1: Yes

Reviewer #2: Yes

**********

**Reviewer #1:**  The research is written in grammatically and scientifically correct language. The statistical analyses were accurate. It would have been preferable for the researcher to include a copy of the questionnaire as an appendix at the end of the research.

The exploratory factor analysis should have been performed before the confirmatory factor analysis.

The scientific references should have been arranged in accordance with APA7.

The discussion of the questionnaire appears to have been insufficient in terms of the theoretical framework underpinning its design.

**Reviewer #2:**  This manuscript tilted "Measurement and Structural Invariance of the Social Determinants of Health Questionnaire (SDH-Q) across Countries" is a cross-sectional study that explores the psychometric properties of the SDH-Q among 860 undergraduate medical students from Malaysia and Nigeria. While the topic is commendable and timely, several core psychometric and methodological concerns must be addressed to strengthen the validity of the findings.

1. Technically Sound (Replication & Rationale): Partly

The comparative approach between Malaysia and Nigeria is intriguing. However, the manuscript would benefit from a more robust theoretical rationale explaining the selection of these specific populations (for example, are there national indicators that underscore the practical importance?).

Furthermore, the authors must clarify the primary scope of this study: Is the objective to validate an instrument specifically for a medical student population, or is it intended for broader community settings to measure SDH? This is crucial in highlighting the importance of the research and in determining a concise research question.

In addition, the absence of a Pilot Study to establish Face Validity is a procedural gap. In cross-cultural psychometrics, pre-testing is essential to ensure that items are interpreted consistently across different linguistic and cultural landscapes.

2. Statistical Analysis and Sampling approach (Appropriateness & Rigor): Partly

The integrity of statistical outcomes is inseparable from the quality of the data inputs. Moving directly to a full-scale analysis without a pilot study introduces a high risk of measurement error. Thus, if the items were misinterpreted by participants, the resulting statistical models will be mathematically flawed regardless of their complexity.

Furthermore, there is a significant Sample-Concept Mismatch, as the inclusion criteria appear to be misaligned with the study's broader objectives. In addition, targeting medical undergraduate students to measure SDH tends to capture "academic perceptions" rather than the direct lived experiences of vulnerable populations, especially those who are most affected by these determinants. Without a preliminary validation to ensure cross-cultural equivalence between Malaysia and Nigeria, the statistical comparisons remain speculative and lack the necessary rigor for a conclusive psychometric study. The authors acknowledge information bias but there is a need to specifically address social desirability bias, which could be prevalent among medical students responding to psychosocial constructs.

4. Intelligible Fashion & Standard English: Partly

The manuscript's narrative is generally clear, but the introduction requires better thematic alignment. Specifically, the information regarding Sustainable Development Goals (SDGs) should not be presented as general background; instead, it must be strategically linked to the scale’s constructs and its field application. For instance, the authors should condense the broad descriptions of SDGs to focus exclusively on those most relevant to the study (e.g., SDG 3: Good Health and Well-being and SDG 10: Reduced Inequalities). This would allow more space to discuss the practical significance of the scale, specifically how its results can inform field-level interventions and evidence-based policymaking in Malaysia and Nigeria. From this perspective, the introduction needs to bridge the gap between global goals and the specific psychometric utility of the tool in identifying social health inequities.

The research question in the introduction lacks the necessary psychometric rigor and precision required to guide the validation process. It is vital for the authors to explicitly state the primary inquiry of the study early on, as this forms the foundation for the subsequent methodology and results. Moreover, there is a logical inconsistency in the introduction’s rationale. The authors justify the need for this study by citing Sabo et al. (2024) (P 5, paragraph 2, line 1; information cited from the reference [27]); however, the data collection for the current study was undertaken in 2023 (P 5, study design ( paragraph 1, line 5)). The authors should clarify this timeline or provide the original sources that informed the study's inception.

Final recommendation:

Decision: Major revision.

The manuscript addresses a timely and significant topic. However, it requires major revisions to align with rigorous academic standards. The methodology is compromised by the absence of a pilot study and misalignment between the target sample and the research objectives.

Furthermore, the authors need to clarify the logical inconsistency regarding the study's timeline and the references used. For the paper to be accepted, it is essential to demonstrate that the measurement tool is valid across different cultures and to better account for potential response bias. These improvements are crucial to ensure the validity and reliability of the findings.

**********

what does this mean?). If published, this will include your full peer review and any attached files.

If you choose “no”, your identity will remain anonymous but your review may still be made public.

**Do you want your identity to be public for this peer review?**  For information about this choice, including consent withdrawal, please see our Privacy Policy

Reviewer #1: No

Reviewer #2: **Yes:**  Amani Kubitary

**********

[NOTE: If reviewer comments were submitted as an attachment file, they will be attached to this email and accessible via the submission site. Please log into your account, locate the manuscript record, and check for the action link "View Attachments". If this link does not appear, there are no attachment files.]

To ensure your figures meet our technical requirements, please review our figure guidelines: https://journals.plos.org/plosone/s/figures

You may also use PLOS’s free figure tool, NAAS, to help you prepare publication quality figures: https://journals.plos.org/plosone/s/figures#loc-tools-for-figure-preparation.

NAAS will assess whether your figures meet our technical requirements by comparing each figure against our figure specifications.

# Author comment: Author response to Decision Letter 1

**DOI:** 10.1371/journal.pone.0356884.r002

14 Jul 2026

Editor comments:

thanks for submitting your article to PLOS ONE. Reviewers comments are provided below. Please address them with mainly the addition of theoretical formwork for the study questionnaire to be expanded in the introduction.

Response: Thank you for your valuable comments and for the reviewers' constructive feedback. In response to your recommendation, we have substantially expanded the theoretical framework underpinning the Social Determinants of Health Questionnaire (SDH-Q) in the Introduction section. Specifically, we provided a more detailed description of the Commission on Social Determinants of Health (CSDH) framework, the conceptual basis of the structural and intermediary determinants of health, and the rationale for how these constructs are operationalized in the SDH-Q. We also strengthened the justification for examining measurement and structural invariance across Malaysia and Nigeria. In addition, we carefully addressed all reviewer comments and have detailed our responses in the accompanying rebuttal letter. We appreciate the opportunity to revise our manuscript and believe that these revisions have strengthened the conceptual foundation and overall quality of the study.

Reviewer 1:

1. The research is written in grammatically and scientifically correct language. The statistical analyses were accurate. It would have been preferable for the researcher to include a copy of the questionnaire as an appendix at the end of the research.

Response: We appreciate the reviewers’ comments and suggestions. We have included the questionnaire as a supporting information file during the submission process, in accordance with the journal’s requirements.

2. The exploratory factor analysis should have been performed before the confirmatory factor analysis.

Response: We thank the reviewer for this valuable comment. Exploratory Factor Analysis (EFA) was not conducted in the present study because the SDH-Q had already been developed and validated by previous authors, who established its factor structure through both EFA and Confirmatory Factor Analysis (CFA). The primary objective of this study was to evaluate the measurement and structural invariance of the SDH-Q across Nigeria and Malaysia rather than to re-explore its underlying factor structure. Also, we have added results of content and face validity in lines 163 – 179, and 253 – 259.

3. The scientific references should have been arranged in accordance with APA7.

Response: We thank the reviewer for this suggestion. However, the references in the present study were done in accordance with the journal's requirement, which is the Vancouver reference style.

4. The discussion of the questionnaire appears to have been insufficient in terms of the theoretical framework underpinning its design.

Response: We thank the reviewer for this valuable comment. We have now added more information on the theoretical development of the SDH-Q in lines 395 - 405.

Reviewer 2:

This manuscript tilted "Measurement and Structural Invariance of the Social Determinants of Health Questionnaire (SDH-Q) across Countries" is a cross-sectional study that explores the psychometric properties of the SDH-Q among 860 undergraduate medical students from Malaysia and Nigeria. While the topic is commendable and timely, several core psychometric and methodological concerns must be addressed to strengthen the validity of the findings.

1. The comparative approach between Malaysia and Nigeria is intriguing. However, the manuscript would benefit from a more robust theoretical rationale explaining the selection of these specific populations (for example, are there national indicators that underscore the practical importance?).

Response: We thank the reviewer for this valuable comment. We have now added more information on the theoretical rationale for testing the measurement and structural invariance of the SDH-Q across Nigeria and Malaysia in our study (lines 94 – 117).

2. The authors must clarify the primary scope of this study: Is the objective to validate an instrument specifically for a medical student population, or is it intended for broader community settings to measure SDH? This is crucial in highlighting the importance of the research and in determining a concise research question.

Response: We thank the reviewer for this valuable comment. We have now added more information on the primary scope of the SDH-Q in lines 155 – 161.

3. The absence of a Pilot Study to establish Face Validity is a procedural gap. In cross-cultural psychometrics, pre-testing is essential to ensure that items are interpreted consistently across different linguistic and cultural landscapes.

Response: We thank the reviewer for this valuable comment. In the present study, we assessed the content and face validity of the SDH-Q among Malaysian experts and students, respectively, as these validity assessments had already been conducted and reported in the original SDH-Q development study involving Nigerian experts and students. The results were initially omitted to maintain a concise manuscript. However, we have now added the findings from the content and face validity assessments conducted in the current study and have also provided the corresponding results from the original Nigerian validation study for comparison and completeness (lines 163 – 179, and 253 – 259).

4. The integrity of statistical outcomes is inseparable from the quality of the data inputs. Moving directly to a full-scale analysis without a pilot study introduces a high risk of measurement error. Thus, if the items were misinterpreted by participants, the resulting statistical models will be mathematically flawed regardless of their complexity.

Response: We thank the reviewer for this valuable comment. Exploratory Factor Analysis (EFA) was not conducted in the present study because the SDH-Q had already been developed and validated by previous authors, who established its factor structure through both EFA and Confirmatory Factor Analysis (CFA). The primary objective of this study was to evaluate the measurement and structural invariance of the SDH-Q across Nigeria and Malaysia rather than to re-explore its underlying factor structure. Also, we have added results of content and face validity in lines 163 – 179 and 253 – 259.

5. The authors acknowledge information bias but there is a need to specifically address social desirability bias, which could be prevalent among medical students responding to psychosocial constructs.

Response: We thank the reviewer for this valuable comment. We have now included this in the study limitation in lines 473 – 476.

6. The manuscript's narrative is generally clear, but the introduction requires better thematic alignment. Specifically, the information regarding Sustainable Development Goals (SDGs) should not be presented as general background; instead, it must be strategically linked to the scale’s constructs and its field application. For instance, the authors should condense the broad descriptions of SDGs to focus exclusively on those most relevant to the study (e.g., SDG 3: Good Health and Well-being and SDG 10: Reduced Inequalities). This would allow more space to discuss the practical significance of the scale, specifically how its results can inform field-level interventions and evidence-based policymaking in Malaysia and Nigeria.

Response: We thank the reviewer for this valuable comment. We have made amendments to the introduction as suggested by the reviewer in lines 94 – 117.

7. Moreover, there is a logical inconsistency in the introduction’s rationale. The authors justify the need for this study by citing Sabo et al. (2024) (P 5, paragraph 2, line 1; information cited from the reference [27]); however, the data collection for the current study was undertaken in 2023 (P 5, study design ( paragraph 1, line 5)). The authors should clarify this timeline or provide the original sources that informed the study's inception.

Response: Response: We thank the reviewer for this valuable comment. We have revised and corrected the data collection period in the manuscript. The previously reported dates were included due to an oversight, as the authors had referred to information from their study published in 2024. The updated data collection dates have now been accurately reflected and supported by the ethical approval extension letter, which has been provided as a supplementary file.

Attachment Submitted filename: rebuttal document.docx

# Peer review: Decision Letter 1

**DOI:** 10.1371/journal.pone.0356884.r003

**Authors:** Taher Hatahet

10 Aug 2026

Measurement and Structural Invariance of the Social Determinants of Health Questionnaire (SDH-Q) across Countries.

PONE-D-26-22540R1

Dear Dr. Kueh,

We’re pleased to inform you that your manuscript has been judged scientifically suitable for publication and will be formally accepted for publication once it meets all outstanding technical requirements.

Within one week, you’ll receive an e-mail detailing the required amendments. When these have been addressed, you’ll receive a formal acceptance letter and your manuscript will be scheduled for publication.

An invoice will be generated when your article is formally accepted. Please note, if your institution has a publishing partnership with PLOS and your article meets the relevant criteria, all or part of your publication costs will be covered. Please make sure your user information is up-to-date by logging into Editorial Manager at Editorial Manager® and clicking the ‘Update My Information' link at the top of the page. For questions related to billing, please contact billing support.

If your institution or institutions have a press office, please notify them about your upcoming paper to help maximize its impact. If they’ll be preparing press materials, please inform our press team as soon as possible -- no later than 48 hours after receiving the formal acceptance. Your manuscript will remain under strict press embargo until 2 pm Eastern Time on the date of publication. For more information, please contact onepress@plos.org.

Kind regards,

Taher Hatahet, Ph.D

Academic Editor

PLOS One

Additional Editor Comments (optional):

Reviewers' comments:

Reviewer's Responses to Questions

**Comments to the Author**

Reviewer #2: All comments have been addressed

**********

2. Is the manuscript technically sound, and do the data support the conclusions?

Reviewer #2: Yes

**********

3. Has the statistical analysis been performed appropriately and rigorously?

Reviewer #2: Yes

**********

4. Have the authors made all data underlying the findings in their manuscript fully available?

The PLOS Data policy

Reviewer #2: Yes

**********

5. Is the manuscript presented in an intelligible fashion and written in standard English?

Reviewer #2: Yes

**********

Reviewer #2: (No Response)

**********

what does this mean?). If published, this will include your full peer review and any attached files.

If you choose “no”, your identity will remain anonymous but your review may still be made public.

**Do you want your identity to be public for this peer review?**  For information about this choice, including consent withdrawal, please see our Privacy Policy

Reviewer #2: **Yes:** Amani Kubitary

**********

# Editor report: Acceptance letter

**DOI:** 10.1371/journal.pone.0356884.r004

**Authors:** Taher Hatahet

PONE-D-26-22540R1

PLOS One

Dear Dr. Kueh,

I'm pleased to inform you that your manuscript has been deemed suitable for publication in PLOS One. Congratulations! Your manuscript is now being handed over to our production team.

At this stage, our production department will prepare your paper for publication. This includes ensuring the following:

* All references, tables, and figures are properly cited

* All relevant supporting information is included in the manuscript submission,

* There are no issues that prevent the paper from being properly typeset

You will receive further instructions from the production team, including instructions on how to review your proof when it is ready. Please keep in mind that we are working through a large volume of accepted articles, so please give us a few days to review your paper and let you know the next and final steps.

Lastly, if your institution or institutions have a press office, please let them know about your upcoming paper now to help maximize its impact. If they'll be preparing press materials, please inform our press team within the next 48 hours. Your manuscript will remain under strict press embargo until 2 pm Eastern Time on the date of publication. For more information, please contact onepress@plos.org.

You will receive an invoice from PLOS for your publication fee after your manuscript has reached the completed accept phase. If you receive an email requesting payment before acceptance or for any other service, this may be a phishing scheme. Learn how to identify phishing emails and protect your accounts at https://explore.plos.org/phishing.

If we can help with anything else, please email us at customercare@plos.org.

Thank you for submitting your work to PLOS One and supporting open access.

Kind regards,

PLOS ONE Editorial Office Staff

on behalf of

Dr. Taher Hatahet

Academic Editor

PLOS One
